# A Systematic Review and Meta-Analysis Estimating the Expected Dropout Rates in Randomized Controlled Trials on Yoga Interventions

**DOI:** 10.1155/2016/5859729

**Published:** 2016-06-16

**Authors:** Holger Cramer, Heidemarie Haller, Gustav Dobos, Romy Lauche

**Affiliations:** ^1^Department of Internal and Integrative Medicine, Kliniken Essen-Mitte, Faculty of Medicine, University of Duisburg-Essen, Am Deimelsberg 34a, 45276 Essen, Germany; ^2^Australian Research Centre in Complementary and Integrative Medicine (ARCCIM), University of Technology Sydney, 15 Broadway, Ultimo, Sydney, NSW 2007, Australia

## Abstract

A reasonable estimation of expected dropout rates is vital for adequate sample size calculations in randomized controlled trials (RCTs). Underestimating expected dropouts rates increases the risk of false negative results while overestimating rates results in overly large sample sizes, raising both ethical and economic issues. To estimate expected dropout rates in RCTs on yoga interventions, MEDLINE/PubMed, Scopus, IndMED, and the Cochrane Library were searched through February 2014; a total of 168 RCTs were meta-analyzed. Overall dropout rate was 11.42% (95% confidence interval [CI] = 10.11%, 12.73%) in the yoga groups; rates were comparable in usual care and psychological control groups and were slightly higher in exercise control groups (rate = 14.53%; 95% CI = 11.56%, 17.50%; odds ratio = 0.82; 95% CI = 0.68, 0.98; *p* = 0.03). For RCTs with durations above 12 weeks, dropout rates in yoga groups increased to 15.23% (95% CI = 11.79%, 18.68%). The upper border of 95% CIs for dropout rates commonly was below 20% regardless of study origin, health condition, gender, age groups, and intervention characteristics; however, it exceeded 40% for studies on HIV patients or heterogeneous age groups. In conclusion, dropout rates can be expected to be less than 15 to 20% for most RCTs on yoga interventions. Yet dropout rates beyond 40% are possible depending on the participants' sociodemographic and health condition.

## 1. Introduction

Attrition, that is, the loss of participants during the course of a study, is a potential threat to internal and external validity in randomized controlled trials (RCTs) [1]; and the underestimation of the size of attrition may severely affect the confidence in the results of a study by increasing the risk in type II errors (false negative results) [2]. Overestimating dropout rates on the other hand will result in overly large sample sizes, raising both ethical and economic issues: unnecessary large numbers of participants might be exposed to a potentially ineffective or even dangerous intervention [3], not to mention the enhanced study expenses related to overly large sample sizes. Given limited available funds, this is probably an even more complex problem in complementary and integrative medicine than in conventional medicine [4]. Yoga not only is among the most commonly used [5] but also is one of the most commonly studied complementary and integrative therapies, with more than 50 randomized controlled trials being published each year now [6].

Study design and patients' baseline characteristics were the most common factors associated with attrition in clinical trials on exercise interventions. Relative to an active comparator, the use of waiting list designs may be detrimental to the attrition rate in the control group [7]. Furthermore, pretreatment physical fitness, depressive symptoms, and increased fatigue level seem to produce higher dropout rates [8, 9] as well as a lower educational level of the participants [10]. Factors specifically associated with attrition in yoga trials have not been identified yet; therefore, dropout rates for yoga trials are mainly estimated based on personal experience or rules of thumb [11]. In order to provide reliable estimates for expected dropout rates in future yoga trials, this systematic review aimed to systematically assess and meta-analyze the reported dropout rates in previously published RCTs on yoga interventions and to analyze their associations with study characteristics.

## 2. Methods

This systematic review was based on a previously published bibliometric analysis that descriptively summarized characteristics of RCTs on yoga interventions [6]. The paper is in line with the Preferred Reporting Items for Systematic Reviews and Meta-Analyses (PRISMA) guidelines [12], unless otherwise indicated.

### 2.1. Eligibility Criteria

#### 2.1.1. Types of Studies

RCTs were eligible. No language restrictions were applied; if necessary, language experts were consulted.

#### 2.1.2. Types of Participants

Studies on all types of participants were eligible. No restrictions were made regarding sociodemographic characteristics or health status.

#### 2.1.3. Types of Interventions

Studies were eligible if they compared yoga interventions to one or more nonyoga interventions or untreated control groups. No restrictions were applied regarding the tradition, length, frequency, or duration of the studied yoga programs. The specific yoga techniques included in the intervention were not restricted as long as the intervention was based on yoga theory and/or traditional yoga practices. Studies allowing individual cointerventions were eligible while studies where yoga was part of a multimodal intervention were excluded.

#### 2.1.4. Types of Outcomes

Studies were eligible if they at least reported the dropout rate for the yoga intervention group.

### 2.2. Literature Search Methods

The literature search comprised four electronic databases from their inception through February 12, 2014 (MEDLINE/PubMed, Scopus, IndMED, and the Cochrane Library) and was constructed around search terms for “yoga” and a filter for retrieving randomized controlled trials [6]. The complete search strategy is shown in Table 1. The reference lists of identified original articles or reviews and the tables of contents of the* Journal of Yoga & Physical Therapy* and the* International Scientific Yoga Journal SENSE* were searched manually for additional eligible studies. Abstracts identified during literature search were screened independently by two review authors; and potentially eligible articles were then read in full by two review authors to determine whether they actually met the eligibility criteria.

### 2.3. Data Extraction

Study and participant characteristics (country of origin, medical conditions, gender, and age groups), intervention characteristics (duration, specific yoga techniques used), and control group characteristics (type of control intervention) were extracted from the included studies independently by two authors using a standardized data extraction form. Dropout rates for the yoga groups and (if available) for the control groups were extracted independently by two authors.

### 2.4. Statistical Analysis

Data were analyzed using a standardized Microsoft Excel (version 12.3.5, Microsoft, Redmond, USA) spreadsheet [13] to calculate prevalence rates and standard errors. The Review Manager software package (version 5.2, Nordic Cochrane Centre, Copenhagen, Denmark) was used to conduct the meta-analysis on the basis of random effects to estimate weighted dropout rates with 95% confidence intervals (95% CIs) for the yoga groups and (if available) for the control groups. Subgroup analyses of dropout rates in the yoga groups were conducted for (1) study origin, (2) medical condition, (3) gender, (4) age group, (5) specific yoga techniques used, and (6) study duration. Subgroup differences were assessed by testing for heterogeneity across subgroups [14] using the *I* ^2^ statistics as a measure of the percentage of variability in effect estimates from the different subgroups that is due to genuine subgroup differences rather than chance. The Chi^2^ test was further used and a *p* value ≤ 0.10 was regarded to indicate significant heterogeneity (see below).

Additionally, odds ratios (OR) with their 95% CIs were calculated to compare dropout rates between the yoga groups and specific control groups. Statistical heterogeneity between studies was analyzed using the *I* ^2^ statistics, a measure of how much variance between studies can be attributed to differences between studies rather than chance. The magnitude of heterogeneity was categorized as (1) *I* ^2^ = 0–24%: low heterogeneity; (2) *I* ^2^ = 25–49%: moderate heterogeneity; (3) *I* ^2^ = 50–74%: substantial heterogeneity; and (4) *I* ^2^ = 75–100%: considerable heterogeneity [14]. The Chi^2^ test was used to assess whether differences in results are compatible with chance alone. Given the low power of this test when only few studies or studies with low sample size are included in a meta-analysis, a *p* value ≤ 0.10 was regarded to indicate significant heterogeneity [14, 15].

## 3. Results

### 3.1. Study Characteristics

**Figure 1:**
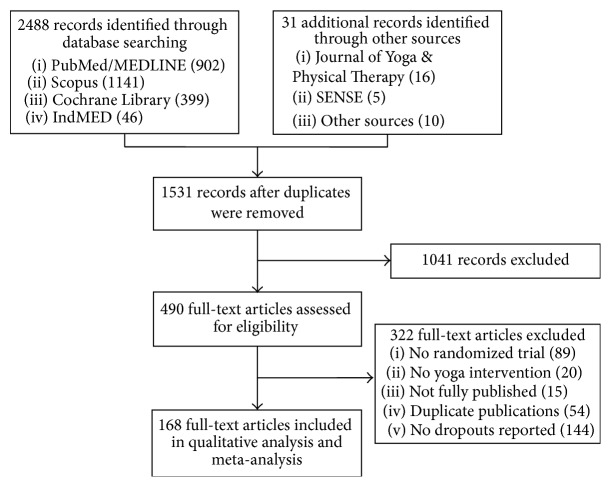
Flow chart of the results of the literature search.

Out of 312 located yoga RCTs, a total of 168 RCTs reporting dropout rates were included (Figure 1). Sixty-six RCTs (39.3%) originated from North America, 3 (1.8%) from South America, 20 (11.9%) from Europe, 69 (41.1%) from Asia, and 10 (6.0%) from Australia. While 47 RCTs (28.0%) included healthy participants, 121 (72.0%) included patients with medical conditions, mainly psychiatric (22 RCTs, 13.1%), musculoskeletal (21 RCTs, 12.5%), cardiovascular (16 RCTs, 9.5%), or oncological (16 RCTs, 9.5%) conditions. Most RCTs included both male and female (106 RCTs, 63.1%) or only female (49 RCTs, 29.2%) participants, only adult participants (82 RCTs, 48.8%), or mixed groups of adults and elderlies (67 RCTs, 39.9%). Median yoga group size was 30 with a range of 8 to 206. Control groups had median sample sizes of 39, 27, and 30 with ranges from 8 to 166, 5 to 204, and 10 to 99 for exercise, usual care, and psychological interventions, respectively.

Regarding yoga interventions, yoga postures, breathing techniques, and meditation were used in 144 (85.7%), 130 (77.4%), and 86 RCTs (51.2%), respectively. 44 (26.2%), 89 (53.0%), and 35 (20.8%) RCTs used intervention durations of less than 8 weeks, 8 to 12 weeks, and more than 12 weeks, respectively.

### 3.2. Estimated Dropout Rates

**Figure 2:**
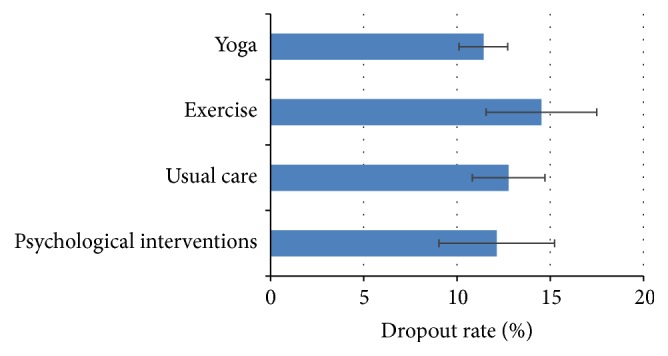
Overall estimated dropout rates (±95% confidence interval) for yoga and control interventions.

Based on the 168 RCTs, overall dropout rate in the yoga groups was 11.42% (95% CI = 10.11%, 12.73%) (Figure 2). Dropout rates were similar in yoga compared to usual care (100 RCTs; rate = 12.77%; 95% CI = 10.82%, 14.72%; OR = 0.92; 95% CI = 0.79, 1.08) or compared to psychological control groups (34 RCTs; rate = 12.13%; 95% CI = 9.03%, 15.22%; OR = 0.86; 95% CI = 0.60, 1.22) but slightly lower in yoga compared to exercise control groups (41 RCTs; rate = 14.53%; 95% CI = 11.56%, 17.50%; OR = 0.82; 95% CI = 0.68, 0.98) (Figure 2, Table 2).

Dropout rates in the yoga groups did not differ between RCTs of different origin (*p* = 0.14; Table 3) but were higher for RCTs on patients with medical conditions (rate = 12.48%; 95% CI = 10.48%, 14.13%) than for RCTs on healthy participants (rate = 9.34%; 95% CI = 10.48%, 14.13%; *p* = 0.02; Table 5). Regarding medical conditions, dropout rates differed strongly based on the specific condition (*p* < 0.01, Table 4), ranging from 0.83% (95% CI = −2.90%, 4.55%) for patients with digestive diseases to 22.20% (95% CI = 4.30%, 40.09%) for HIV patients. Likewise, dropout rates differed based on gender and age group with the highest dropout rates in RCTs including female participants only and in RCTs including both adolescents and adults (Table 4).

Regarding intervention characteristics, dropout rates were higher for RCTs that included yoga postures (12.00%, 95% CI = 10.53%, 13.46% versus 7.22%, 95% CI = 4.32%, 10.11; *p* > 0.01) and/or meditation (12.67%, 95% CI = 10.75%, 14.60% versus 10.07%, 95% CI = 8.25%, 11.89; *p* = 0.05) (Table 5) and gradually increased with intervention duration from 9.42% (95% CI = 6.93%, 11.91%) for a duration of less than 8 weeks to 15.23% (95% CI = 11.79%, 18.68%) for a duration of more than 12 weeks (*p* = 0.03; Table 5).

## 4. Discussion

### 4.1. Summary of Evidence

In this systematic review of 168 randomized controlled trials, on average 11.42% of all trial participants within the yoga groups dropped out during the trial. The dropout rates were mainly comparable to those in the other trial groups including usual care or psychological interventions; and they were slightly smaller compared to those in the exercise control groups. Differences in dropout rates were further found for patients with medical conditions compared to healthy participants and between patients with different medical conditions; for comparisons based on participants' gender and age; and for comparisons based on yoga's components and intervention duration.

Several findings deserve attention. First, the dropout rate in yoga groups at postintervention was relatively small. Given the rule of thumb that up to 20% of dropout during a trial can be considered acceptable [11], the majority of trials did not exceed this rate. The rate was further comparable to the rate in usual care or psychological interventions within the same trials precluding bias due to unbalanced dropouts in trial groups. Dropout rates are however slightly smaller than in exercise control groups indicating less attrition in the yoga study arms. Even though this analysis cannot provide sufficient explanation for this difference, it might be related to the recruitment process and the patients' preferences for either intervention.

As for the patients' characteristics, this analysis found that the dropout rate in healthy participants was significantly lower than in participants with medical conditions. And the condition itself may limit regular participation and adherence to yoga classes; we also found major differences between different patient subgroups. Patients diagnosed with oncological diseases or HIV and pregnant women, for example, had almost twice the dropout rate compared with patients with musculoskeletal disorders with upper borders of 95% CIs up to 40%. The severity of the medical conditions must therefore be considered an important factor when calculating the sample size for a trial [16]. The very low dropout rates in studies on digestive diseases [17, 18] may be explained by gender and age characteristics of the examined samples that included only male participants and adolescents.

Trials on only male participants had very low dropout rates while those including females only had more than four times as many. Trials with males only might however have used different settings, for example, the army forces [19] or workplace [20, 21]. These environments may have provided a specific structure and daily routine that increased compliance and adherence compared to other trials. Studies on females only also included those trials with pregnant women with high dropout rates, for example, due to pregnancy complications or onset of labor, thereby raising the average dropout rate for women in general. Moreover, most cancer-related trials were on female breast cancer patients [22–29] and associated with a relatively high dropout rate. It is therefore crucial to bear in mind the special circumstances for each trial when planning the respective study.

Furthermore, trials with children and adolescents only had very low attrition rates. This may be related to the settings of those trials, with studies being conducted in schools and colleges providing a suitable structure and daily routine for such trial. Interestingly, trials including both adolescents and adults had substantially higher dropout rates; however, the rate was calculated based on two trials only [30, 31]. So while this finding remains difficult to interpret, it seems advisable not to plan yoga interventions for adolescents and adults together.

Last but not least, the intervention characteristics played an important role in dropout rates. As for the intervention length, there was a clear association between the length of the intervention period and the dropout rate with almost double the attrition in trials over 12 weeks compared to trials up to 8 weeks. Such increase in dropout rates with increasing trial length is a common occurrence and can be observed in other nonyoga trials as well [16, 32]. As for the yoga components, trials incorporating yoga postures and meditation had higher dropout rates than those without those components. This is in line with findings that yoga-associated adverse events are often associated with specific yoga postures [33], although more adverse events have been reported for breathing techniques than for meditation.

The findings of this analysis may benefit future yoga research in many ways. For one it may present researchers with an estimate of expected dropout rates for future RCTs on yoga, taking into account several intervention or participant related factors. Findings from a large number of trials can thereby lead to a more accurate estimation of expected dropout rates than personal experience or rules of thumb can.

They may further enable researchers in specific scenarios to prepare for expected high dropout rates and to discuss strategies to successfully retain participants in the trial. Such strategies have been evaluated before in a variety of settings [34, 35].

Analyzing and comparing dropout rates during the trial can also provide information about the acceptability and safety of an intervention [32]. A recent meta-analysis however did not find any particular safety concerns associated with yoga, and rates of adverse events were comparable to that of exercise control interventions [36].

This study also faces some limitations. Only 168 of 312 RCTs (53.8%) could be included in the analysis; the other trials had to be excluded as they did not provide sufficient information about dropouts and withdrawal. Furthermore, only a minority of studies sufficiently described detailed reasons for dropouts. In order to judge whether the study may be biased (attrition bias), such information is as vital as the total number of dropouts. Due to the paucity of data, it was also not possible to analyze interactions between the study and participants' characteristics. Therefore, information on expected dropouts can only be considered a rough estimation.

Finally, researchers should be aware that there are many other factors influencing dropout rates, for example, the general setting (facility access), the study conditions (personnel, reimbursement of travel costs), and soft factors such as empathy of doctors and nurses.

### 4.2. Conclusion

Dropout rates usually can be expected to not exceed 15 to 20% in the majority of RCTs on yoga interventions. Yet dropout rates beyond 40% are possible depending on the participants' sociodemographic and health condition. This meta-analysis can serve as a guideline for sample size calculation in future RCTs on yoga interventions.

## Figures and Tables

**Table 1:** Search strategy.

PubMed	
#1	Yoga[MeSH Terms]
#2	Yoga^*∗*^[Title/Abstract] OR Yogi^*∗*^[Title/Abstract]
#3	#1 OR #2
#4	Randomized Controlled Trial[Publication Type]
#5	Random^*∗*^[Title/Abstract]
#6	#4 OR #5
#7	#3 AND #6

Scopus	
#1	TITLE-ABS-KEY(yoga^*∗*^) OR TITLE-ABS-KEY(yogi^*∗*^)
#2	TITLE-ABS-KEY(random^*∗*^)
#3	#1 AND #2

Cochrane Library	
#1	MeSH descriptor: [Yoga] explode all trees
#2	Yoga^*∗*^:ti, ab, kw (Word variations have been searched)
#3	Yogi^*∗*^:ti, ab, kw (Word variations have been searched)
#4	#1 OR #2 OR #3
#5	MeSH descriptor: [Randomized Controlled Trial] explode all trees
#6	Random^*∗*^:ti, ab, kw (Word variations have been searched)
#7	#5 OR #6
#8	#4 AND #7

IndMED	
#1	(Yoga OR Yogic) and (Random OR Randomized OR Randomised OR Randomly)

**Table 2:** Differences in estimated dropout rates between yoga and control interventions. CI: confidence interval; OR: odds ratio.

Comparison	OR [95% CI]	*p* value
Yoga versus exercise	0.82 [0.68, 0.98]	0.03
*Heterogeneity: Chi* ^2^ = 38.50*, df* = 40 (*p* = 0.54*), I* ^2^ = 0%		
Yoga versus usual care	0.92 [0.79, 1.08]	0.31
*Heterogeneity: Chi* ^2^ = 133.84*, df* = 102 (*p* = 0.02*), I* ^2^ = 24%		
Yoga versus psychological interventions	0.86 [0.60, 1.22]	0.40
*Heterogeneity: Chi* ^2^ = 57.09*, df* = 34 (*p* = 0.008*), I* ^2^ = 40%		

**Table 3:** Estimated dropout rates for yoga interventions as a function of study characteristics (country of origin). CI: confidence interval.

Country of origin	Number of studies	Rate [95% CI]
North America	66	11.79 [9.67, 13.92]
South America	3	3.07 [−3.73, 9.86]
Europe	20	13.79 [8.93, 18.64]
Asia	69	11.37 [9.35, 13.40]
Australia	10	11.69 [5.26, 18.11]
*Test for subgroup differences: Chi* ^2^ = 6.85*, df* = 4 (*p* = 0.14*), I* ^2^ = 41.6%		

**Table 4:** Estimated dropout rates for yoga interventions as a function of participant characteristics (medical condition, gender, and age groups). CI: confidence interval.

Condition	Number of studies	Rate [95% CI]
Medical conditions	121	12.48 [10.83, 14.13]
Healthy	47	9.34 [7.16, 11.51]
*Test for subgroup differences: Chi* ^2^ = 5.09*, df* = 1 (*p* = 0.02*), I* ^2^ = 80.3%		

Condition, specific	Number of studies	Rate [95% CI]

Musculoskeletal	21	7.54 [4.53, 10.55]
Cardiovascular	16	16.50 [11.03, 21.96]
Psychiatric	22	11.60 [7.38, 15.83]
Oncologic	16	18.04 [11.12, 24.96]
Pulmonary	9	12.95 [5.63, 20.28]
Neurological	10	12.87 [7.22, 18.52]
Endocrine	6	9.17 [1.75, 16.60]
Urogenital	10	11.94 [9.00, 14.89]
Digestive	2	0.83 [−2.90, 4.55]
Pregnancy	7	24.22 [12.38, 36.07]
HIV	2	22.20 [4.30, 40.09]
*Test for subgroup differences: Chi* ^2^ = 47.67*, df* = 10 (*p* < 0.00001*), I* ^2^ = 79.0%		

Gender	Number of studies	Rate [95% CI]

Male only	10	3.16 [−0.17, 6.50]
Female only	49	14.19 [11.36, 17.02]
Mixed gender	106	10.98 [9.37, 12.59]
*Test for subgroup differences: Chi* ^2^ = 25.44*, df* = 2 (*p* < 0.00001*), I* ^2^ = 92.1%		

Age groups	Number of studies	Rate [95% CI]

Children and adolescents only	7	5.62 [2.44, 8.80]
Adolescents and adults	2	26.61 [11.37, 41.85]
Adults only	82	11.20 [9.33, 13.06]
Elderlies only	10	10.06 [4.62, 15.50]
Adults and elderlies	67	12.86 [10.60, 15.12]
*Test for subgroup differences: Chi* ^2^ = 17.74*, df* = 4 (*p* = 0.001*), I* ^2^ = 77.4%		

**Table 5:** Estimated dropout rates for yoga interventions as a function of intervention characteristics (yoga postures, breathing techniques, meditation, and duration). CI: confidence interval.

Yoga postures	Number of studies	Rate, 95% CI
Including postures	144	12.00 [10.53, 13.46]
Not including postures	21	7.22 [4.32, 10.11]
*Test for subgroup differences: Chi* ^2^ = 8.32*, df* = 1 (*p* = 0.004*), I* ^2^ = 88.0%		

Breathing techniques	Number of studies	Rate, 95% CI

Including breathing techniques	130	11.80 [10.27, 13.33]
Not including pranayama	34	10.63 [7.87, 13.40]
*Test for subgroup differences: Chi* ^2^ = 0.53*, df* = 1 (*p* = 0.47*), I* ^2^ = 0%		

Meditation	Number of studies	Rate, 95% CI

Including meditation	86	12.67 [10.75, 14.60]
Not including meditation	78	10.07 [8.25, 11.89]
*Test for subgroup differences: Chi* ^2^ = 3.70*, df* = 1 (*p* = 0.05*), I* ^2^ = 73.0%		

Intervention duration	Number of studies	Rate, 95% CI

Less than 8 weeks	44	9.42 [6.93, 11.91]
8–12 weeks	89	11.18 [9.42, 12.94]
More than 12 weeks	35	15.23 [11.79, 18.68]
*Test for subgroup differences: Chi* ^2^ = 7.21*, df* = 2 (*p* = 0.03*), I* ^2^ = 72.3%		
